# Edward Jenner: The Pioneer of Vaccination and His Enduring Legacy in Modern Medicine

**DOI:** 10.7759/cureus.68805

**Published:** 2024-09-06

**Authors:** Pooja Mary Vaishali, Nisha Boopathy

**Affiliations:** 1 Department of Community Medicine, Saveetha Medical College and Hospital, Saveetha Institute of Medical and Technical Sciences, Saveetha University, Chennai, IND

**Keywords:** biographies, edward jenner, historical vignettes, medical innovation, medical stories

## Abstract

Edward Jenner’s work in the latter part of the 18th century laid the groundwork for contemporary vaccination techniques and represented a crucial moment in the battle against contagious diseases. Born in 1749 in Berkeley, Gloucestershire, Jenner received early medical training under the guidance of John Hunter, a distinguished British surgeon. Although variolation offered some immunity to smallpox, it was a hazardous procedure that could result in severe illness or even death. It was within the context of limited and hazardous medical practices that Jenner made his revolutionary observation that milkmaids who had contracted cowpox, a relatively mild illness, appeared to be immune to smallpox. This local folklore piqued Jenner’s interest, leading him to investigate the potential of cowpox as a safer alternative to variolation. His work paved the way for the development of vaccines for other infectious diseases, transforming public health and establishing a foundation for modern immunology. The smallpox vaccine became a crucial element of public health initiatives, ultimately leading to the global eradication of the disease by the late 20th century. Jenner’s contributions have saved countless lives and represent a testament to the enduring influence of his work on global health. His pioneering efforts laid the groundwork for vaccines that protect us today, solidifying his place as one of the most influential figures in medical history.

## Introduction and background

Edward Jenner was born on May 17, 1749, in Berkeley, Gloucestershire, England, into a family of modest means. His father was a clergyman, and his mother contributed to a stable yet ordinary upbringing for Jenner. Despite these unassumed beginnings, Jenner exhibited an early fascination with nature and medicine, which shaped his future career. At the age of 13, Jenner embarked on formal medical education through an apprenticeship with a local surgeon. This early exposure to the medical field laid the foundation for his subsequent achievements. His unwavering commitment to studying medicine led him to London, where he furthered his education under the mentorship of John Hunter, a prominent surgeon and leading figure in British medicine at the time. Hunter’s influence on Jenner was profound; he nurtured Jenner’s innate curiosity and emphasized the importance of empirical observation and rigorous experimentation. This mentorship was crucial to shaping Jenner’s scientific approach and critical thinking [1]. Upon completing his apprenticeship, Jenner returns to Berkeley to establish his own medical practice. His work in Berkeley was marked by an increasing interest in local medical phenomena, particularly in the connection between cowpox and smallpox. Local folklore suggested that milkmaids who contracted cowpox, a disease with symptoms much milder than those of smallpox, seemed to be immune to smallpox. This observation captured the attention of Jenner and set the stage for his groundbreaking research. During the late 1700s, smallpox was a devastating disease with an exceptionally high mortality rate, causing widespread suffering and death. Jenner’s curiosity about the relationship between cowpox and smallpox motivated him to propose that cowpox could be utilized as a means of conferring immunity against smallpox. This idea was revolutionary because it challenged the conventional medical practices of the time and aimed to address a critical public health issue [2]. Jenner’s experiments involved inoculating individuals with matter taken from cowpox sores, which he referred to as vaccination. This approach was a notable departure from existing practices, which relied on variolation, a technique that entailed infecting individuals with smallpox to induce immunity. Jenner’s method proved to be a safer and more effective means of preventing smallpox, ultimately leading to the development of the first efficient smallpox vaccine. The success of Jenner’s vaccination method had significant implications for medicine and public health. By demonstrating that immunity could be elicited through the use of cowpox, Jenner not only provided a means to combat smallpox but also established a foundation for the development of vaccines for other diseases. His pioneering work laid the groundwork for modern immunology and vaccination practices, revolutionizing the field of medicine and saving countless lives around the world.

## Review

The pre-vaccination era

**Figure 1:**
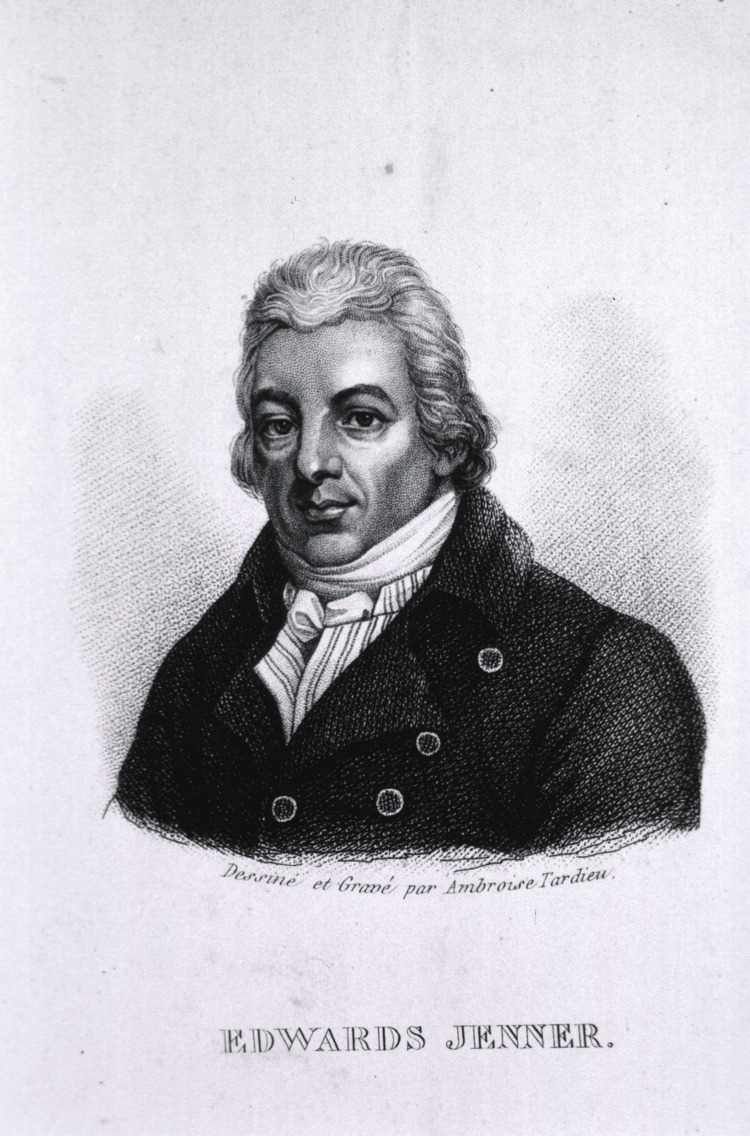
Images from the History of Medicine (IHM) contributor(s): Tardieu, Ambroise, artist. Source: National Library of Medicine [5].

Jenner was an English surgeon and discoverer of a vaccine for smallpox (Figure 1). Before Jenner’s groundbreaking discovery, medical knowledge regarding infectious diseases was exceedingly rudimentary. During the 18th century, the scientific foundation of medical practices was relatively narrow, leading to a lack of effective measures to prevent or control diseases that ravaged the population. Smallpox, a disease that elicited widespread fear and panic, is among the most destructive illnesses. Characterized by fever, body aches, and the appearance of pus-filled blisters, smallpox is caused by the variola virus, with mortality rates as high as 30%. Survivors often endure long-lasting scarring or blindness, making smallpox a threat to global health [3]. Prior to the introduction of vaccination, various approaches were taken to curb the transmission of smallpox with limited success. One such technique was variolation, an ancient form of inoculation that was practiced in numerous regions around the world, including China, India, the Middle East, and Africa. This method involved deliberately exposing a person to smallpox-infected material obtained from a lesion, typically by applying it to a scratch on the skin [4]. While variolation often resulted in a milder form of the disease and subsequent immunity, it was a risky procedure, as it could lead to a severe case or even prove fatal.

Smallpox epidemics

Smallpox epidemics were a recurrent and daunting reality before the advent of vaccination. The disease was indiscriminate, impacting people of all ages, social classes, and regions. Smallpox was particularly lethal in densely populated urban areas where it rapidly spread from person to person. One of the most notable smallpox epidemics occurred in Europe during the early 18th century, causing considerable mortality and societal disruption [6]. The fear of smallpox was so pervasive that it affected various aspects of daily life, including social practices, travel, and political decisions. In response to these epidemics, governments and communities sought various methods to control the spread of the disease. Quarantines were often imposed on affected households, and variolation had become increasingly common as a preventive measure despite its risks [7]. However, these efforts were only partially effective, and the frequent recurrence of smallpox epidemics emphasized the need for a more reliable method of disease prevention. The limitations of existing methods, coupled with the severe impact of smallpox on public health, created a climate of desperation and innovation, in which Edward Jenner’s work would soon emerge as a beacon of hope. Jenner’s introduction of vaccination not only offered a safer alternative to variolation but also provided the first opportunity to control and eventually eradicate smallpox [8].

Edward Jenner’s development of the smallpox vaccine was influenced by his keen observation and innovative experimental techniques. In the 18th century, Jenner observed that milkmaids who had contracted cowpox, a milder illness, seemed immune to smallpox, a severe and often deadly disease. Based on his observations, Jenner hypothesized that cowpox could be used to protect against smallpox. In 1796, Jenner conducted a groundbreaking experiment in which he inoculated an eight-year-old boy, James Phipps, with material from a cowpox lesion. After the boy developed mild cowpox, Jenner exposed him to smallpox, and Phipps did not contract the disease, demonstrating the protective effect of cowpox against smallpox (Jenner, 1798). Jenner’s method involved careful observation, documentation, and repetition to validate his findings, laying the groundwork for modern vaccination practices [9]. Jenner’s findings, published in “An Inquiry into the Causes and Effects of Variolae Vaccinae” in 1798, provided a comprehensive account of his experiments and the theoretical basis for vaccination. Initially, the medical community was skeptical and debated the validity of his work, but over time, the efficacy of vaccination became evident through further trials and observations. Jenner’s innovative approach not only revolutionized preventive medicine but also established the foundation for the development of vaccines against various infectious diseases, marking a significant advancement in public health [10].

Jenner’s experimental methods

Edward Jenner’s groundbreaking method of creating the smallpox vaccine was built upon empirical observation and practical experimentation. He observed that dairymaids who had contracted cowpox, a mild endemic illness that affects cattle, seemed to be immune to smallpox, a highly virulent and often fatal disease [11]. Jenner (1796) conducted a series of pivotal experiments to test this hypothesis. He inoculated an eight-year-old boy named James Phipps with material taken from a cowpox lesion on the dairymaid’s hand (Jenner, 1798). After the boy developed a mild cowpox, Jenner exposed him to smallpox. Remarkably, Phipps did not contract the disease, demonstrating the protective effect of cowpox. To ensure reliability, Jenner’s method involved careful subject selection, meticulous documentation of disease progression, and repeated trials. He also conducted similar experiments on other individuals to further validate his findings, which was an essential step in establishing the effectiveness of vaccination [12]. Jenner’s experimental technique was a pioneering approach for testing and validating a medical intervention, laying the foundation for modern vaccine development by demonstrating that a less virulent pathogen could confer immunity to a more dangerous one.

Jenner’s publication of the findings

Edward Jenner’s groundbreaking work, “An Inquiry into the Causes and Effects of Variolae Vaccinae,” was published in 1798. This influential paper provides a detailed account of Jenner’s research and experiments on the smallpox vaccine. He documented his observations, experimental methods, and results of his trials with cowpox (Jenner, 1798). The publication introduced the concept of vaccination using cowpox to protect against smallpox and provided a theoretical framework for this novel preventive measure. The reception of Jenner’s findings was initially mixed, with some in the medical community skeptical and resistant to vaccination. However, over time, as more evidence accumulated supporting Jenner’s findings and the effectiveness of vaccination became increasingly apparent, the medical community began to accept and adopt these methods. Jenner’s work ultimately had a profound and lasting impact on public health, setting the stage for vaccine development and the field of immunology [13].

Jenner’s observations about milkmaids and cowpox

Dr. Edward Jenner’s groundbreaking discovery emerged from his observations during his medical practice in the rural county of Gloucestershire. He noticed a noteworthy pattern: local milkmaids who had contracted cowpox, a relatively benign disease caused by the vaccinia virus, seemed to be immune to smallpox, a severe and often fatal illness, with a high mortality rate. Cowpox, characterized by lesions on the hands and arms, is significantly less dangerous than smallpox, which could result in disfiguring scars or even death [14]. This observation was influenced by local folklore, which suggested a protective effect against smallpox among those who had previously contracted cowpox. Jenner’s insight was rooted in this empirical observation but advanced by his hypothesis that cowpox exposure could indeed confer immunity to smallpox [15]. Jenner’s approach was pioneering because it offered a safer alternative to the common practice of variolation, which involved deliberately infecting individuals with material from smallpox sores. Variolation carried significant risks, including severe illness and death, and was not always effective. Jenner hypothesized that cowpox, being a less virulent disease, could serve as an effective preventive measure without severe complications associated with variolation. This idea was radical, yet promising, as it suggested a way to protect against smallpox without the high risks of traditional methods (Jenner, 1798). Jenner’s observation of milkmaids and application of his hypothesis through experimentation marked the beginning of modern vaccination and transformed public health by providing a safer method to achieve immunity [16].

The experiment: Jenner’s first vaccination experiment on James Phipps

In 1796, Jenner conducted an experiment that significantly impacted modern vaccination methods. He chose James Phipps, an eight-year-old boy from Berkeley, Gloucestershire, as the subject of his experiment to test his hypothesis that cowpox could provide immunity against smallpox, a highly lethal disease with a high mortality rate and severe complications at the time. Cowpox, a milder disease affecting cattle, was a promising method of protection. Jenner’s experimental procedure began with the inoculation of Phipps using material taken from a cowpox lesion on the hands of a local dairymaid, Sarah Nelmes. He made a small incision and carefully inserted this material into the arm of Phipps, which was a groundbreaking approach to testing the protective properties of cowpox [17]. Over the next few days, Phipps developed a minor case of cowpox, characterized by a few lesions at the inoculation site and a normal and expected reaction. To confirm this hypothesis further, Jenner conducted a second test on Phipps. Approximately six weeks after the initial cowpox inoculation, Jenner intentionally exposed Phipps to smallpox by inoculating him with smallpox material. The results of this study indicated that past exposure to cowpox protected Phipps against smallpox, validating Jenner’s hypothesis. This trial was pivotal in the creation of the smallpox vaccine and represented a departure from the hazardous practice of variolation. Jenner’s technique, which utilized cowpox, presented a less hazardous and more effective alternative to variolation. The success of this investigation not only provided empirical evidence for the use of cowpox in vaccination but also sparked a significant change in the approach to disease prevention. Jenner’s work demonstrated the potential of vaccination as a safer and more efficient method of immunization than existing techniques, laying the groundwork for future advancements in immunology and public health. This groundbreaking experiment continues to be a significant event in medical history, marking the dawn of the vaccination era and the progression of preventive medicine.

Jenner’s impact on contemporary medicine

Edward Jenner’s groundbreaking work in the late 18th century has had a profound and enduring impact on contemporary medicine, particularly in the fields of immunology and public health. His discovery that a vaccine could prevent smallpox, a highly contagious and deadly disease, paved the way for vaccine development against a wide range of infectious diseases. Jenner’s innovative approach involved using cowpox, a less harmful virus, to provide immunity against smallpox and establishing fundamental principles of vaccination that continue to underpin modern vaccine development [18]. These principles have laid the foundation for vaccination as a safe and effective means of disease prevention. Jenner’s method of inducing immunity through vaccination has since become the mainstay of preventive medicine. The success of Jenner’s smallpox vaccine inspired further research and development of vaccines for various other diseases such as rabies, polio, measles, mumps, and rubella. The development of these vaccines has significantly reduced the incidence of these diseases and saved countless lives. The principles established by Jenner’s work also extend to the development of modern vaccines, such as the human papillomavirus vaccine, which protects against cervical cancer, and hepatitis vaccines, which protect against liver disease. More recently, Jenner’s principles have become crucial for the rapid development of vaccines against COVID-19. mRNA vaccines, such as those developed by Pfizer-BioNTech and Moderna, exemplify an advanced application of vaccination principles, in which genetic material was employed to stimulate an immune response without utilizing a live virus. Edward Jenner’s influence extends beyond scientific advancements to encompass global health strategies. Vaccination remains a key component of public health efforts worldwide and is essential for the control and eradication of infectious diseases. Initiatives, such as the Global Polio Eradication Initiative and routine childhood vaccination schedules, reflect the profound impact of Jenner’s work. Vaccination programs have significantly reduced and, in some cases, eliminated the incidence of diseases that once caused widespread morbidity and mortality Jenner’s pioneering work continues to save millions of lives annually, demonstrating the enduring power of scientific discovery and its impact on global health. In summary, Edward Jenner’s contributions to medicine have had far-reaching effects on contemporary practices in immunology and public health. His establishment of vaccination principles laid the groundwork for vaccine development against a wide array of infectious diseases. The ongoing application of these principles in vaccine development highlights Jenner’s lasting legacy and underscores the importance of his work in advancing human health and combating diseases [19-21]

Accolades and monuments

Edward Jenner’s significant contributions to the field of medicine have been widely recognized and honored through various accolades, monuments, and memorials that pay tribute to his pioneering work on vaccination. His enduring legacy is memorialized by several statues and memorials, showcasing his profound impact on public health. One of the most notable tributes is the bronze statue by William Calder Marshall, which was originally installed in Trafalgar Square, London, in 1858. This statue was later relocated to Kensington Gardens, highlighting Jenner’s esteemed position in British medical history. Another significant monument stands in Jenner’s birthplace of Berkeley, Gloucestershire, where a statue has been erected to honor his groundbreaking contributions and his deep connection to the local community. These statues serve not only as physical reminders of Jenner’s achievements but also as symbols of his lasting influence on the field of medicine. Moreover, in addition to physical monuments, Jenner’s impact extended beyond the prestigious awards and honors that recognized his pioneering work. In 1789, he was elected as a Fellow of the Royal Society, an acknowledgment of his earlier work in natural history and a testament to his scientific contributions. This recognition was a precursor to the widespread acclaim that Jenner would receive for his development of the smallpox vaccine, which has saved countless lives and continues to be an essential tool in public health efforts worldwide. The esteem in which the scientific community held his work was evidenced by the numerous accolades and distinctions he received during his lifetime, and this recognition continued posthumously through the establishment of institutions and awards named in his honor. One such institution was the Jenner Institute at the University of Oxford, which was founded in 2005. This institute is at the forefront of vaccine research and development and embodies Jenner’s legacy by focusing on creating vaccines against a wide array of infectious diseases [22]. The Jenner Institute represents a modern extension of Jenner’s original work, continuing his mission to protect and improve public health through vaccinations. The institute’s commitment to advancing vaccine science underscores the enduring relevance of Jenner’s contribution to contemporary medicine. In addition to the Jenner Institute, numerous medical schools, hospitals, and awards worldwide bear Jenner’s name, reflecting his profound influence in the medical field. These institutions and awards perpetuate Jenner’s legacy and honor his role in the advancement of immunology and vaccine development [23]. The widespread naming of facilities and awards after Jenner highlighted the global recognition of his work and its ongoing impact on public health strategies. Jenner’s contributions to medicine have been immortalized not only through physical monuments and institutional honors but also through the continued application of his principles in modern medicine. His innovative approach to vaccination laid the groundwork for the field of medicine.

## Conclusions

Edward Jenner’s pioneering work in the field of vaccination was a landmark achievement in the history of medicine, representing a significant turning point in disease prevention and paving the way for modern immunology. His innovative approach, which utilized cowpox to confer immunity against smallpox, not only provided a safer alternative to the traditional method of variolation but also laid the groundwork for the development of vaccines against numerous other contagious diseases. The lasting impact of Jenner’s legacy is evident in the countless lives saved, which serves as a testament to the paramount importance of scientific advancements in promoting global health.
